# Cisplatin resistance alters ovarian cancer spheroid formation and impacts peritoneal invasion

**DOI:** 10.3389/fcell.2025.1450407

**Published:** 2025-02-05

**Authors:** Lydia C. Powell, Marcos Quintela, David W. James, Emenike Onyido, David Howard, Kadie Edwards, Jordan L. Turney, Charlotte R. Morgan, Jenny Worthington, Nicole Williams, Alexander Dulebo, Heiko Haschke, Deyarina Gonzalez, R. Steven Conlan, Lewis W. Francis

**Affiliations:** ^1^ Swansea University Medical School, Faculty of Medicine, Health and Life Sciences, Swansea, United Kingdom; ^2^ AxisBio Discovery Services, Londonderry, United Kingdom; ^3^ Bruker Nano GmbH, Berlin, Germany

**Keywords:** ovarian cancer, spheroids, biophysics, invasion, atomic force microscopy, cisplatin

## Abstract

Epithelial ovarian cancer (EOC) is an aggressive and lethal gynaecologic malignancy due to late diagnosis and acquired resistance to chemotherapeutic drugs, such as cisplatin. EOC metastasis commonly occurs through the extensive dissemination of multicellular aggregates, formed of cells originally shed from the primary ovarian tumour, within the peritoneal cavity. However, little is known about how cisplatin resistance (CR) alters the biophysical properties of EOC multicellular aggregates and how this impacts metastasis. In this interdisciplinary study, light and atomic force microscopy was used, alongside quantitative gene and protein expression analysis, to reveal distinct differences in the biophysical properties of CR spheroids, which correlated with altered protein expression of plasminogen activator inhibitor-1 (PAI-1) and Tenascin-C. CR SKOV3 spheroids (IC50: 25.5 µM) had a significantly greater area and perimeter and were less spherical, with a reduced Young’s modulus, (p < 0.01) compared to parental (P) SKOV3 spheroids (IC50: 5.4 µM). Gene expression arrays revealed upregulation of genes associated with cell adhesion, extracellular matrix (ECM) and epithelial-to-mesenchymal transition (EMT) in CR spheroids, while immunofluorescence assays demonstrated increased protein expression of PAI-1 (p < 0.05; implicated in cell adhesion) and reduced protein expression of Tenascin-C (p < 0.01; implicated in elasticity) in CR spheroids compared to P spheroids. Furthermore, the CR spheroids demonstrated altered interactions with a surface that mimics the peritoneal lining post mesothelial clearance (Matrigel). CR spheroids were significantly less adhesive with reduced disaggregation on Matrigel surfaces, compared to P spheroids (p < 0.05), while CR cells were more invasive compared to P cells. The combined characterisation of the biophysical and biological roles of EOC multicellular aggregates in drug resistance and metastasis highlight key proteins which could be responsible for altered metastatic progression that may occur in patients that present with cisplatin resistance.

## Introduction

Epithelial ovarian cancer (EOC) is an aggressive and lethal gynaecologic malignancy (Liao et al., 2014; Nowacka et al., 2021), with only a 5-year survival rate of 46% after diagnosis (Doherty et al., 2017; Quintela et al., 2023). Treatment for EOC normally involves a surgical cytoreduction followed by first line chemotherapy based on taxane (Paclitaxel) and platinum (such as Cisplatin; Nowacka et al., 2021). EOC is frequently diagnosed at an advanced stage of the disease, due to asymptomatic or vague symptoms, and often acquires therapeutic resistance to chemotherapy, contributing to the high death to incidence rate observed in patients (Lengyel, 2010; Lheureux et al., 2019; Nowacka et al., 2021).

EOC patients commonly present with metastatic disease within the peritoneal cavity (Lengyel, 2010; Nowacka et al., 2021). EOC metastasis occurs both through direct extension from the primary tumour site (either from the ovary, fallopian tube or the peritoneum) to neighbouring organs (bowel and bladder), as is common in other types of cancer, and uniquely through cell seeding within the peritoneal cavity (Lengyel, 2010). Intraperitoneal metastasis occurs when cells or cell clusters shed from the primary ovarian tumour into the peritoneal cavity and adhere to one another to form multicellular aggregates. These aggregates are then transported by the physiological movement of the ascites, where they then facilitate extensive dissemination of cancer cells across the mesothelial-lined peritoneum, leading to peritoneal metastasis (Lengyel, 2010; Liao et al., 2014; Al Habyan et al., 2018; van Baal et al., 2018).

The biophysical properties of cancer cells have previously been linked to cell survival, malignancy and metastatic ability (Lekka, 2016; Deng et al., 2018; Stylianou et al., 2018; Andolfi et al., 2019; Abidine et al., 2021; Mahajan et al., 2021), where biophysical changes occur in the context of significant alterations in the cellular gene expression profiles (Ansardamavandi et al., 2020; Lu and Anvari, 2020; Toubhans et al., 2020; Mahajan et al., 2021). Specifically, cancer cells have been shown to be more elastic than non-malignant types, with increased cell deformability which is thought to facilitate metastatic progression (Mierke, 2021). Within EOC multicellular aggregates, the cancer cells are densely packed and embedded within a network of extracellular matrix (ECM; such as collagen, laminin and fibronectin), which allows for oxygen and nutrient diffusion gradients within the structure, resulting in cell hypoxia and glycolysis (Chowanadisai et al., 2016; Vyas et al., 2019). Therefore, the biophysical properties of these multicellular aggregates result not only from the mechanical properties of the cancer cells alone (in relation to their cytoskeleton and plasma membrane) but also from the whole multicellular aggregate structure, due to the complex crosslinking between cell adhesion molecules and the ECM network (Blumlein et al., 2017; Andolfi et al., 2019; Boot et al., 2021). Indeed, these mechanical forces are thought to be integral to multicellular aggregate development, through cell-packing density and architecture (Boot et al., 2021) and are postulated to be key parameters in dissemination and metastasis (Mierke, 2021). Little is known, however, about how drug resistance alters the biophysical properties of EOC multicellular aggregates formed within the peritoneal cavity and how these properties impact subsequent metastasis (Vyas et al., 2019).

Spheroids are *in vitro* 3D multicellular aggregate model systems that express an intermediate complexity between the 2D *in vitro* and *in vivo* models (Han et al., 2021; Paradiso et al., 2021). Spheroids are widely used to mimic features of *in vivo* tumours, such as their physiological responses, internal architecture, drug resistance mechanisms, ECM deposition, gene expression patterns and cell-cell and ECM-cell interactions (Costa et al., 2016; Guillaume et al., 2019; Vyas et al., 2019; Boot et al., 2021). Previous studies have demonstrated that ovarian cancer spheroids form robust structures, with paclitaxel drug resistance and higher presence of apoptotic cells (Matte et al., 2016; Tofani et al., 2020). Therefore, spheroids are perfectly placed to mimic the 3D structure of multicellular aggregates formed during ovarian cancer metastasis, allowing fundamental biophysical insights to be gained into aggregate formation, metastasis and chemotherapeutic resistance (Boot et al., 2021; Han et al., 2021).

In this study, we examined the morphological and mechanical properties of spheroids derived from parental (P) and cisplatin resistant (CR) EOC SKOV3 cell lines. In addition, we quantified the gene and protein expression associated with the ECM and cytoskeleton within P and CR EOC spheroids. The influence of cisplatin resistance on EOC spheroid adhesion, disaggregation and invasion into a peritoneum basement membrane mimic was then examined to uncover the biophysical mechanisms of EOC chemotherapeutic resistance in metastasis. This greater mechanistic understanding may be beneficial in highlighting key proteins involved in EOC intraperitoneal metastasis and may aid in the development of new targeted treatment strategies for EOC (Liao et al., 2014; Chowanadisai et al., 2016; Hedemann et al., 2018).

## Experimental

### Cell culture

SKOV3 cell line used was originally purchased from ATCC^®^ (Manassas, Virginia, United States) and was used as the parental cell line (P). An acquired cisplatin-resistant (CR) SKOV3 cell line was derived from the P cell line by AxisBio discovery systems (Howard et al., 2022). SKOV3 cells were maintained (37°C, 5% CO_2_) in McCoy’s media supplemented with 10% fetal bovine serum (FBS) and 1% antibiotic-antimycotic solution in plastic culture vessels (25 cm^2^, 75 cm^2^). Cells were supplemented with full serum media every 2 days and passaged when confluent. Only cells passaged two or more times were used for this study. The CR SKOV3 cell line was exposed to cisplatin (20 μL at 0.5 μg/mL) once a week (in 75 cm^2^ culture vessels) to ensure selective pressure on the cell line to maintain resistance. The cells were thoroughly washed before each experiment to ensure no cisplatin was present unless stated.

### Spheroid culture

P and CR spheroids were produced in a 96-well Ultra-Low attachment (ULA) surface microplate (Corning TM 4520). For the Western blot assay, spheroids were formed in a 96-well plate (167425, ThermoFisher Scientific) coated with 2% agarose (50 µL per well) which was sterilised with UV light before use. For most assays, unless otherwise stated, 5000 cells were added per well in 200 µL McCoy’s media supplemented with 10% FBS and 1% antibiotic-antimycotic solution. The plate was then placed into an incubator (37°C, 5% CO_2_) over 24–96 h to allow for spheroid formation.

### 2D IC50 cell viability

Cell viability was monitored using Realtime-Glo™ MT Cell Viability Assay (RT-Glo; Promega, G9712). SKOV3 cells were seeded into a 96-well microtiter plate with white opaque walls (Porvair Krystal, 214006), with seeding densities of 500 cells for SKOV3 variants per well in 100 µL. Cells were allowed to adhere overnight, where prior to treatment cells were washed twice with phosphate buffered saline (PBS). Treatments were prepared at double concentration (2X) in media containing charcoal-stripped, heat inactivated FBS only. Media only was used as a negative control and used for any normalisation. NanoLuc^®^ enzyme and MT cell viability substrate were diluted in stripped media such that the final concentration was 2X (viability reagents) of that provided in the Promega Kit as recommended. Finally, 50 µL of 2X treatments and 2X viability reagents were added such that final concentrations were 1X. Plate was incubated at 37°C and luminescence readings were taking using a FLUOstar Omega microplate reader pre-heated to 37°C at 0-, 24-, 48-, 72- and 96 h time points. Finally, results were normalised to negative controls and plotted using GraphPad Prism v. 10.1.2.

### Spheroid viability

Spheroids (2 × 10^3^ cells) were seeded in ULA 96-well plates for 48 h prior to treatment with 5.4 µM of cisplatin (IC50 value of P cells) for a further 48 h. Spheroid viability was quantified using Celltiter-Glo^®^ 3D as per manufacturers instruction (Promega, G9682), an endpoint assay that contains a potent lytic agent that disrupts spheroid structures and then quantifies cell viability based on ATP concentration. ATP is utilized by Ultra-Glo™ Luciferase enzyme which causes a bioluminescent signal that can be read via spectrophotometer. Prior to use, the Celltiter-Glo reagent was thawed overnight at 4°C and then left at room temperature (RT) for 1 h before use. Media volume in each well was adjusted to 100 μL and then 100 µL of reagent added. The well plates were shaken for 5 min at 700 rpm and then left to incubate at RT for a further 25 min to allow the luminescent signal to stabilise prior to reading.

### Spheroid morphological analysis

Light microscopy images were taken of the P and CR spheroids produced over 24–98 h. Brightfield images were taken with a Zeiss PrimoVert microscope with a 4x lens before analysis with the open-source AnaSP software (Piccinini, 2015). The AnaSP software characterised the morphological parameters of perimeter, area, sphericity and length of major diameter of these spheroids. For the comparison between P and CR SKOV3 spheroids, 21 spheroids were analysed per cell line from a minimum of three biological repeats.

### Spheroid viability confocal laser scanning microscopy assay

P and CR spheroids were stained with a mixture of three dyes: 2 µM Calcein AM (C1430, Invitrogen), 3 µM Ethidium homodimer-1 (E1169, Invitrogen), 33 µM Hoechst (33342, Invitrogen) for 3 h at 37°C before imaging. The spheroids were placed into 8-well imaging chambers (µ-slide 8-well ibiTreat, Ibidi) in PBS. The spheroids were z-stack imaged with a Zeiss LSM710 confocal laser scanning microscope (CLSM), with a 10x objective (1024 × 1024 pixels), using a 4 µm step size. For the comparison between P and CR SKOV3 spheroids, 12 spheroids were analysed per cell line from a minimum of three biological repeats.

### Atomic force microscopy spheroid mechanical measurements

Cell-Tak (Corning) coated glass coverslips were used for spheroid immobilisation. A coating mixture of 10 µL of Cell-Tak, 285 µL of 0.1 M Sodium Bicarbonate pH 8, and 5 µL of 1 M NaOH was used per slide, then slides were incubated for 1 h (at RT) before being rinsed with deionised water (x2). The spheroids were placed onto the Cell-Tak coated glass coverslips in phenol-red free McCoy’s media (40 μL; HyClone, Cytiva) and incubated for 10 min to allow spheroid attachment to the surface. The slide was transferred to the atomic force microscopy (AFM) stage, after which a further 100 µL of phenol-red free McCoy’s media was added. The mechanical properties of the spheroids were examined using a Bioscope Catalyst AFM (Bruker Instruments; Berlin). The Young’s modulus, indentation depth and the probe adhesion force of the spheroids was achieved using a borosilicate colloidal AFM probe with a sphere diameter of 20 µm and spring constant of 0.35 N/m (NovaScan). Each probe was calibrated for deflection sensitivity and spring constant on a glass slide prior to each measurement. For the force and frequency ramping experiments, four spheroids were analysed for each sample and approximately 10 force curves were acquired from each spheroid. A ramp size of 7 μm, tip speed of 5–30 μm/s and an applied force of 1–20 nN was used. For direct comparison between P and CR SKOV3 spheroids, 24 spheroids were analysed per cell line from a minimum of three biological repeats and approximately 14 force curves were acquired from each spheroid. A ramp size of 7 μm, tip speed of 5 μm/s and an applied force of 10 nN was used for this experiment. The force curves were fitted to the Hertz model and analysed with the Nanoscope analysis software (v1.5, Bruker).

### RNA extraction and gene expression arrays

Total RNA from 3D spheroids was isolated using the RNeasy^®^ Plus Mini Kit (Qiagen, 74136) and reverse transcribed using the high-capacity cDNA reverse transcription kit (Thermo Scientific, 4368814). PCR Arrays (Bio-rad, PrimePCR^™^ PCR Arrays: cytoskeleton remodelling and ECM remodelling) were conducted following manufacturer’s instructions. All PCR array reactions were conducted in a CFX96^™^ real-time PCR detection system (Bio-rad) using iTaq^™^ Universal SYBR^®^ Green supermix (Bio-rad, 1725125). Relative gene expression was determined following the ΔC_t_ method (Yuan et al., 2006) and normalised to an internal reference gene (GAPDH). *t*-test statistical analyses were performed on ΔC_t_ values of two biological replicates.

### IN Cell high content cellular imaging

SKOV3 cells were seeded at a density of 5000 cells per well in black-walled 96 well plates (Ibidi, 89626) and incubated at 37°C and 5% CO_2_. After 24 h, the cells were fixed using 4% paraformaldehyde (PFA; Thermo Fisher Scientific, 28906) in PBS for 15 min and then permeabilised using 0.1% Triton X-100 (Thermo Fisher Scientific, A16046.AE) in PBS for 5 min at RT. The wells were washed with PBS (x1) and then blocked for 1 h at RT using 3% w/v bovine serum albumin (BSA) in PBS. Cells were incubated overnight at 4°C with primary antibodies: anti-E-cadherin (Santa Cruz, Biotechnology, sc-8426), anti-N-cadherin (Abcam, ab18203) anti-vimentin (Santa Cruz Biotechnology, sc-6260) and anti-tenascin C (Santa Cruz Biotechnology, sc-25326) all at 1:200 dilutions in 0.1% BSA/PBS solution. The wells were then washed with PBS (x3). A 0.1% BSA/PBS solution containing fluorescently labelled anti-mouse (Abcam, ab150117) and anti-rabbit (Abcam, ab6564) secondary antibodies (diluted 1:500), and also the counterstains Hoescht 33342 (diluted 1:2000) and phalloidin-Alexa568 (diluted 1:200) were added to each well. After 1 h incubation, wells were washed with PBS (x3) and then promptly imaged using the IN Cell 6000 Analyzer (Molecular Devices; n = 5).

All IN Cell images were manually examined and low-quality images, including out-of-focus or contaminated images were removed from the analysis dataset. Segmentation of cells was performed using Cell Profiler software (Broad Institute; Carpenter et al., 2006). For each image channel an illumination correction function was generated from all corresponding images using the “CorrectIlluminationCalculate” module, selecting the “Regular” option. The location of cell nuclei was identified from the DAPI channel using the “IdentifyPrimaryObjects” module using “minimum cross entropy” to set the intensity threshold. Declumping of clumped nuclei was performed using the “shape” setting. The location of cells was identified from the dsRed (cytoskeleton) channel using the “IdentifySecondaryObjects” module with the “propagation” method. Morphological parameters relating to the shape of nuclei and cells were measured for each individual nucleus and cell using the “MeasureObjectSizeShape” module. Morphological parameters relating to the pixel intensity of markers from each image channel were measured within the regions defined for individual cell and nuclei using the “MeasureIntensity” module. The morphological parameter “form factor”, which is a measure of the shape of the cell, was determined where a value of one indicates a perfect circle, with values < 1 becoming more irregular. Morphological parameter measurements were exported to csv file. Analysis of morphological parameters was performed using R v4.1.2 (R Core Team, 2021). Integrated fluorescence intensity measurements for each marker within the boundaries of each cell were averaged per well. Violin and boxplots for cellular form factor measurements were generated using ggplot2 (Wickham H, 2016).

### Immunofluorescence staining and CLSM imaging of spheroids

Spheroids were fixed and permeabilised with 4% PFA and 1% Triton X in PBS for 3 h at 4°C. The spheroids were washed in PBS (10 min, x3) then dehydrated in an ascending series of ice-cold methanol in PBS (25, 50, 75% and 95%) at 4°C with 20 min incubations. Then, the spheroids were placed into 100% methanol for 1 h before rehydration in the same descending series of ice-cold methanol at 4°C with 20 min incubations. The spheroids were then washed in PBS (10 min, x3) before blocking in PBST (0.1% Triton X in PBS) containing 3% BSA overnight at 4°C. The spheroids were washed in PBST (15 min, x2) and then incubated with the primary antibodies diluted in PBST at 4°C for 72 h. The commercial primary mouse antibodies used for immunofluorescence staining were Vimentin V9 (1:50 dilution; sc-6260, Santa Cruz Biotechnology), Tenascin-C E9 (1:50 dilution; sc-25326, Santa Cruz Biotechnology), E-cadherin (1:50 dilution; ab1416, Abcam), N-cadherin (1:50 dilution; sc-59987, Santa Cruz Biotechnology) and serpine-1/PAI-1 (1:50 dilution; ab66705, Abcam). Spheroids were then rinsed in PBST (15 min, x4), before incubation with secondary antibodies for 24 h at 4°C, with further rinsing in PBST (15 min, x4). The commercial secondary antibodies used were the anti-mouse Alexa Fluor 488 (1:200 dilution; A-11001, Invitrogen) and anti-mouse Texas Red (1:200 dilution; T6390, Invitrogen). Spheroids were counterstained with Hoechst 33342 (1:1000 dilution; Invitrogen) for 25 min before rinsing the spheroids with PBS (15 min, x3). Following the immunofluorescence staining assay, the spheroids were placed into 8-well imaging chambers (µ-slide 8-well ibiTreat, Ibidi) in PBS. The spheroids were z-stack imaged with a Zeiss LSM710 CLSM, with a 10x objective (1024 × 1024 pixels), using a 4 µm step size. Five spheroid biological repeats were analysed per cell line for Vimentin V9, Tenascin-C E9, E-cadherin and N-cadherin staining conditions, while 10 spheroid biological repeats were analysed per cell line for serpine-1/PAI-1.

### Protein blot analysis

48 spheroids were used per condition to generate protein lysates. Radio-immuno precipitation assay (RIPA) buffer (R0278; Sigma-Aldrich) was fortified with HALT™ protease inhibitor cocktail 100X (87786; Thermo Fisher Scientific) to prevent protein degradation, referred to henceforth as RIPA + buffer. Protein extracts were obtained from 48 individual spheroids, where the spheroids were pooled into a single 15 mL falcon tube. Samples were washed twice with dPBS and 70 μL RIPA + buffer added for cell lysis; spheroids that were too buoyant to precipitate independently were centrifuged at 100 rcf for 1 minute. Samples were subjected to three freeze-thaw cycles using dry ice and a water bath set to 37°C followed by the isolates being vortexed every 5 mins for a total of six cycles and left on ice. Isolates were centrifuged in a pre-cooled microfuge at 21,000 rcf for 10 mins at 4°C to pellet cell debris; supernatant was transferred to a new eppendorf and stored at −80°C.

Total protein lysates (30 μg) were resolved on a precast 4%–20% polyacrylamide gels (mini-PROTEAN^®^ TGX stain-free™ gels, 456–8094), transferred and immobilized onto polyvinylidene fluoride (PVDF) membranes (mini format Trans-Blot^®^ Turbo™ transfer pack, 170–4156), incubated for 60 min at RT in blocking solution (TRIS-buffered saline [TBS] containing 5% BSA and 0.1% Tween 20), followed by an overnight incubation in primary antibodies at 4°C (1:1000 dilution). The following antibodies were used: Santa Cruz Biotechnology (Dallas, TX, United States): GAPDH (sc47724), N-cadherin (sc-59987), Tenascin-C E9 (sc-25326), Vimentin V9 (sc-6260) or AbCam (Cambridge, United States): Serpine1/PAI-1 (ab66705), E-cadherin (ab1416). Membranes were then washed three times with TBS-T (TBS with 0.1% Tween 20) and incubated with horseradish peroxidase–conjugated secondary antibodies (ECL anti-rabbit IgG, NA934V or ECL anti-mouse IgG, NA931V; GE Healthcare, United Kingdom) for 1 h (1:2000 dilution). Membranes were incubated for 1 min with Clarity ECL substrate (1:1 dilution of peroxide and luminol reagents) and imaged with a ChemiDoc MP (Bio-Rad) using Chemi High Resolution (signal accumulation mode between 1–120 s) and colorimetric modes. Images were analysed using Image Lab software (Bio-Rad) to obtain adjusted volume intensities. All markers were normalised against housekeeping protein GAPDH.

### Spheroid matrigel adhesion assay

Matrigel growth factor reduced (GFR) membrane matrix (356231; Corning) and DMEM/F12 media (Gibco) were placed on ice at 4°C overnight. The Matrigel was then diluted into DMEM/F-12 media in a 1:4 ratio, before 25 µL of this mixture was added per well into a flat-bottomed 96-well plate (Nunclon Delta Surface, Thermo Scientific) within a sterile environment. The diluted Matrigel mixture was left to set at RT for 1 h before rinsing each well with DMEM/F-12 media (x2). A spheroid was placed into each Matrigel-coated well with McCoy’s media supplemented with 10% FBS and 1% antibiotic-antimycotic solution (150 µL). The plate was placed into the incubator for 3 h (37°C, 5% CO_2_) before being placed onto a rocker for 5 min at 40 rpm. Each well was then rinsed with PBS (x3) before examining the wells under light microscopy (Zeiss PrimoVert microscope with a 4x lens) and counting the remaining number of spheroids attached to the Matrigel surface. For the comparison between P and CR SKOV3 spheroids, 24 spheroids were analysed per cell line from a minimum of three biological repeats.

### Spheroid matrigel disaggregation assay

The Matrigel-coated 96-well plates were produced in the same manner as the Spheroid Matrigel Adhesion assay. Each spheroid was then placed into a Matrigel-coated well with McCoy’s media supplemented with 10% FBS and 1% antibiotic-antimycotic solution (150 µL). A brightfield image of each spheroid was then taken (Zeiss PrimoVert microscope with a 4x lens) before the plate was placed into the incubator (37°C, 5% CO_2_). After 24 h, another brightfield image of each spheroid was taken. The AnaSP software was then used to measure spheroid area before and after 24 h incubation and the % increase in area was calculated. For the comparison between P and CR SKOV3 spheroids, 24 spheroids were analysed per cell line from a minimum of three biological repeats.

### Cell invasion assay

CytoSelect 96-well cell invasion assay with inclusion of ECM basement membrane (CBA-112; Cell BioLabs, Inc) was used in this study. The ECM included in the assay was extracted from the Engelbreth-Holm-Swarm mouse sarcoma which is richly composed of ECM proteins such as laminin and collagen IV, thereby showing close similarity to the extraction process and components of Matrigel. The cell invasion plate was prepared as per manufacturer instructions. 150 μL of McCoy’s media (containing 10% FBS and 1% antibiotic-antimycotic solution) was added to the lower wells in the feeder tray. Then 0.8 × 10^6^ cells/mL were prepared in FBS-free McCoy’s media, with and without the addition of 50 ng/mL VEGF-165 recombinant protein (Invitrogen™), and 100 µL of the cell culture added to the upper well. The plate was incubated for 24 h before processing the plate for cell detachment and staining as per the manufacturer instructions. 150 μL of the stained cell mixture was transferred to a 96-well plate suitable for fluorescence measurement and this plate was read by a fluorescence plate reader (FluoStar Omega, BMG Labtech) at 480/520 nm. For the comparison between P and CR SKOV3 cell lines, a minimum of three biological repeats were assessed.

### Statistical analysis

Statistical analyses were performed using GraphPad Prism v. 9.5 software. Values represent means ± SD (standard deviation). For each test, *P* < 0.05 was considered significant. Normality assessment of the data (Shapiro-Wilk analysis) was performed to assess whether parametric or non-parametric statistical testing was appropriate. Statistical comparisons were assessed either using a *t*-test for parametric data or Mann Whitney test for non-parametric data. Multiple group statistical comparisons were assessed with either with a one-way or two-way analysis of variance (ANOVA) test, with Tukey’s multiple comparison tests. Non-parametric data of multiple groups was statistical compared using the Kruskal–Wallis test and Dunn’s multiple comparison test. Specific tests are noted in each figure legend.

## Results

### Cisplatin resistance alters SKOV3 spheroid morphology

**FIGURE 1:**
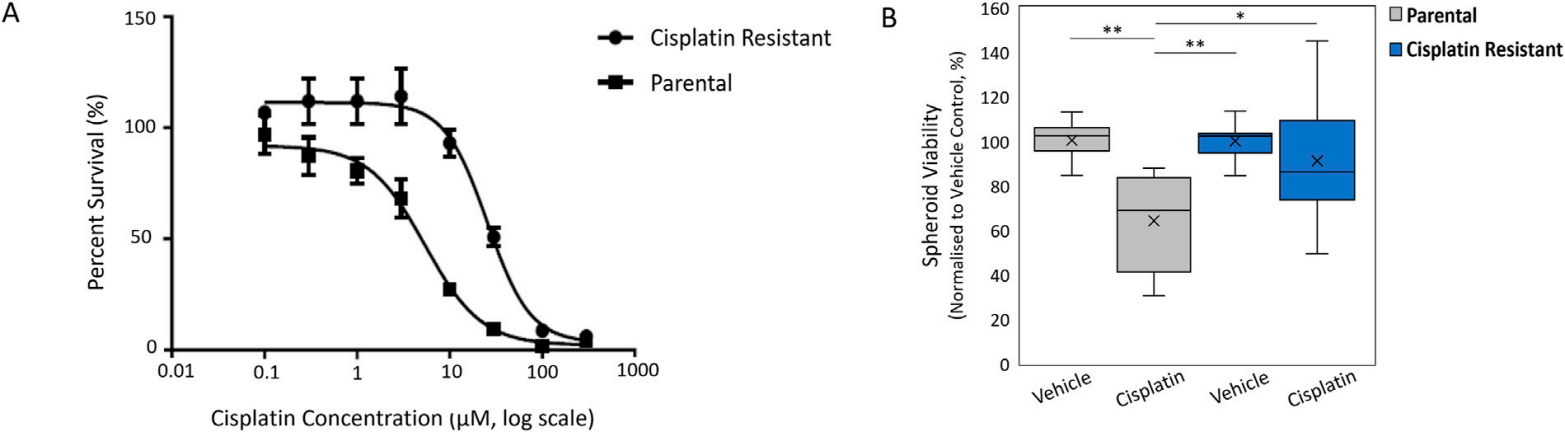
**(A)** Cell viability curves to determine the IC50 value of cisplatin against Parental SKOV3 and Cisplatin-resistant SKOV3 cells. **(B)** Spheroid viability normalized to vehicle control of cisplatin against Parental SKOV3 and Cisplatin-resistant SKOV3 spheroids. Data shown is based on a minimum of three biological repeats (n = 3), statistically analysed as parametric data using one-way ANOVA test with Tukey’s multiple comparison test. Significance given as *p < 0.05, **p < 0.01.

**FIGURE 2:**
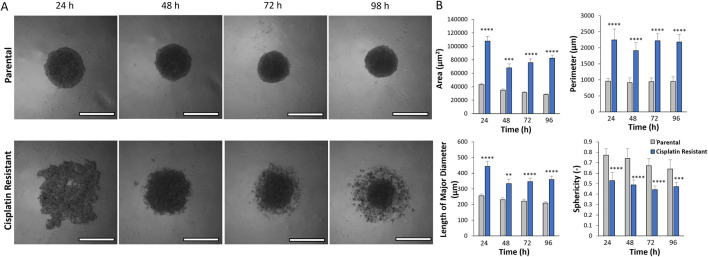
**(A)** Light microscopy images of parental and cisplatin-resistant SKOV3 spheroids formed over 24–98 h (Scale bar 200 µm). **(B)** AnaSP analysis of the spheroid light microscopy images to quantify the area (µm^2^), perimeter (µm), length of the major diameter (µm) and the sphericity (−) of the spheroids. Data is shown as the mean and SD of 21 spheroids and a minimum of three biological repeats (n = 3), statistically analysed as non-parametric data using the Kruskal-Wallis test with Dunn’s multiple comparison test. Significance given as **p < 0.01, ***p < 0.001, ****p < 0.0001.

**FIGURE 3:**
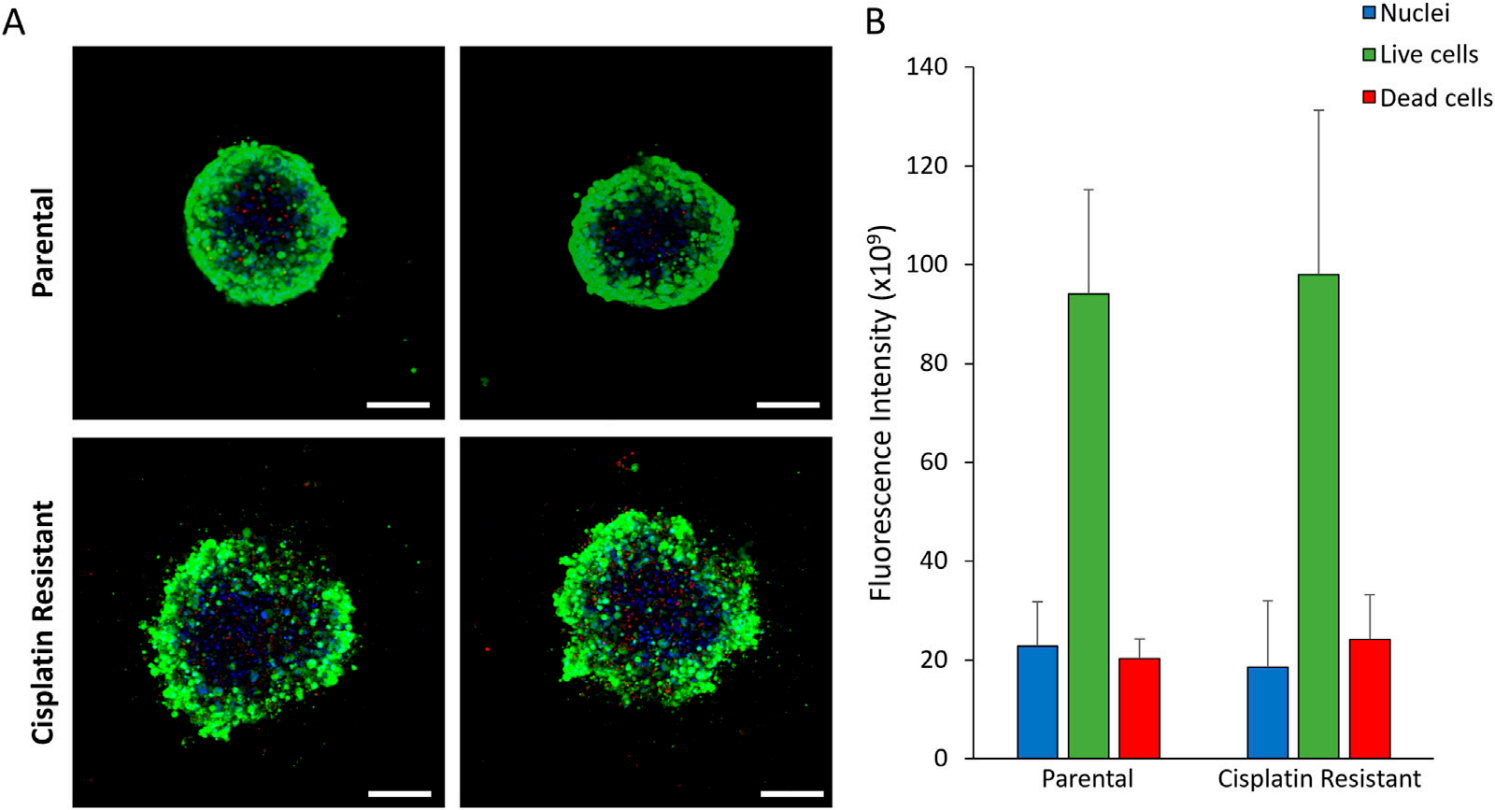
**(A)** CLSM images of 48 h parental and cisplatin-resistant SKOV3 spheroids stained with LIVE/DEAD staining and Hoechst to visualize the nucleus (Scale bar 200 µm). **(B)** Quantification of the fluorescence intensity of the fluorescent markers in the CLSM images. Data is shown as the mean and SD of 12 spheroids and a minimum of three biological repeats (n = 3). The data was statistically analysed as non-parametric data using the Mann Whitney test, however, no significant differences were determined.

The resistance to cisplatin of P and CR SKOV3 cell lines was assessed, with the IC50 values of CR SKOV3 cell line shown to be 5 times more resistant to cisplatin treatment when compared to P cells in 2D culture (IC50: 25.5 µM vs. 5.4 µM, IC25: 46 µM vs. 10 μM, IC75: 13 µM vs. 1.7 µM for CR vs. P cells respectively; Figure 1A). Similarly, the P SKOV3 spheroids, grown for 48 h prior to treatment with 5.4 µM cisplatin for a further 48 h, demonstrated a significant reduction in viability (p < 0.01) which was not apparent in the treated CR spheroids (Figure 1B). To assess the impact of cisplatin resistance on spheroid morphology over time, microscopy images taken at 24 h intervals for a total of 98 h were analysed using AnaSP software (Figure 2). The analysis revealed that for all time points (24–98 h) CR spheroids had a significantly larger area, perimeter and length of major diameter, while exhibiting significantly reduced sphericity when compared to the P spheroids (Figure 2; p < 0.01). CR spheroids at the time points of 72 and 98 h exhibited a more disaggregated cellular morphology which surrounded a denser spheroid core (Supplementary Figure S1). The CR spheroids developed the most compact structure after 48 h compared to the other time-points (Figure 2). CLSM imaging using LIVE/DEAD staining revealed that the changes in spheroid morphology were not due to cellular death at 48 h (Figure 3). However, spheroids developed after 72 and 96 h, possessing a more disaggregated morphology, demonstrated a decrease in fluorescence intensity obtained from live cells (p < 0.01; Supplementary Figure S2). Subsequent experiments used spheroids cultured for 48 h due to their robust, compact structures which is a requirement for sample handling in the following assays.

### Cisplatin resistance alters the mechanical properties of SKOV3 spheroids

AFM offers the advantages of technique sensitivity, spatial resolution (intracellular and intercellular length scales) and high versatility that is required for spheroid mechanical measurements, where AFM has previously proven instrumental in the investigation of cancer cell and tissue mechanics (Doak et al., 2008; Andolfi et al., 2019; Vyas et al., 2019; Boot et al., 2021). To determine the correct system parameters for AFM mechanical measurements on the P and CR spheroids, the indentation force (Supplementary Figure S3) and tip frequency (Supplementary Figure S4) was varied and the spheroid mechanical properties of Young’s modulus, indentation and probe adhesion measured (n = 4 spheroids; Supplementary Tables S1, S2). Colloid probes of 20 µm diameter were used to overcome the nanomechanical heterogeneities on the spheroid surface, resulting in more global measurements (Vyas et al., 2019). Analysis revealed that increasing the indentation force from 1 to 20 nN (Supplementary Figure S3) resulted in a significant increase in Young’s modulus (mean value: 0.92 vs. 1.56 kPa for P spheroids, 0.40 vs. 1.09 kPa for CR spheroids; p < 0.001) and indentation depth (mean value: 828 vs. 2530 nm for P spheroids, 1313 vs. 3170 nm for CR spheroids; p < 0.0001). In addition, while the adhesion of the AFM tip to the P spheroids significantly increased with indentation force (5–20 nN), only the 20 nN indentation force induced a significant increase in probe adhesion to CR spheroids in comparison to the 1 nN force (mean value: 0.07 vs. 0.30 nN for P spheroids, 0.21 vs. 0.51 nN for CR spheroids; p < 0.01). AFM tip frequency, however, did not alter the measurement of Young’s modulus and indentation depth obtained from both the P and CR spheroids and only the P spheroids demonstrated a significant increase in probe adhesion with increasing tip frequency (mean values from 1 to 20 nN: 0.223 vs. 0.476 nN; p < 0.0001; Supplementary Figure S4). These experiments identified suitable system parameters of an indentation force of 10 nN and tip speed of 5 μm/s for the AFM interrogation of the P and CR spheroids.

**FIGURE 4:**
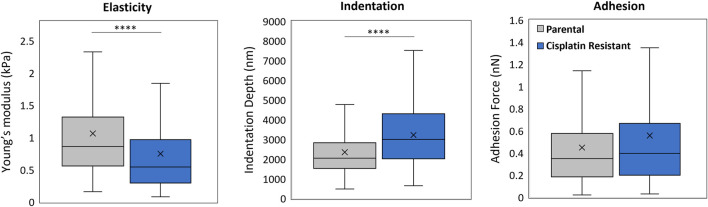
AFM force measurement analysis of 48 h parental and cisplatin-resistant SKOV3 spheroids resulting in measurements of Young’s modulus (kPa), indentation depth (nm) and adhesion (nN). Data is shown as box plots produced from a minimum of 336 curves, 24 spheroids per sample and a minimum of three biological repeats (n = 3), statistically analysed as non-parametric data using Mann Whitney test. Significance given as ****p < 0.0001.

The impact of cisplatin resistance on the mechanical properties of SKOV3 spheroids were measured, where CR SKOV3 spheroids exhibited a significantly reduced Young’s modulus (mean values: 1.06 vs. 0.75 kPa; p < 0.0001) and a significantly increased indentation depth (mean values: 2353 vs. 3226 nm; p < 0.0001) compared to the P spheroids (Figure 4; n = 24 spheroids). No significant difference was observed in probe adhesion between the spheroid samples (p > 0.05).

### Cisplatin resistance alters the expression of ECM and cytoskeleton genes

**FIGURE 5:**
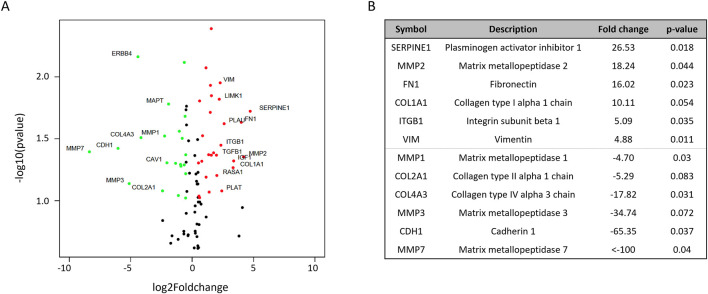
PCR arrays examining differential cytoskeleton and ECM remodeling gene expression between parental and cisplatin-resistant SKOV3 spheroids from two biological repeats **(A)** Volcano plot highlighting the most significantly upregulated (red) or downregulated (green) cytoskeleton and ECM genes. **(B)** List of the six most significantly upregulated and downregulated cytoskeleton and ECM genes, including fold changes and p-value. *t*-test statistical analyses were performed on ΔCt values of two biological replicates (n = 2).

In order to investigate the phenotypical differences in the cellular or spheroid structure (Figures 2, 4) depicted by the mechanical properties of CR spheroids, PCR arrays of cytoskeleton and ECM remodelling target genes were conducted (Figure 5). From a total of 90 queried genes, 41 were found significantly enriched in CR spheroids compared with P spheroids (p < 0.05). Of these 41 genes, 19 were upregulated and 11 downregulated (|Fold-change| > 1.5) (Figure 5A). VIM (Vimentin) and FN1 (fibronectin) expression were significantly upregulated, while CDH1 (cadherin 1), a transmembrane protein that plays a crucial role in intracellular adhesion, was downregulated. During epithelial-to-mesenchymal transition (EMT) in ovarian carcinoma, there is downregulation of E-cadherin expression which is located at cell adherent junctions, upregulation of N-cadherin expression and upregulation in the mesenchymal marker of vimentin (Ray et al., 2023). The gene expression changes measured in the PCR arrays potentially indicate EMT processes occurring during acquisition of cisplatin resistance in EOC aggregates, maybe resulting in increased cellular motility, invasion and metastasis (Lengyel, 2010; Bozhkova and Poryazova-Markova, 2019; Ray et al., 2023). Other genes, linked to cancer cell remodelling/resistance and biomechanics, with significant differential expression were elevated levels of SERPINE1 (Pan et al., 2017) and COL1A1 (collagen type I alpha one chain; An et al., 2020) in CR spheroids, demonstrating fundamental differences between P and CR SKOV3 spheroids (Figure 5B) at the transcriptomic level.

### Cisplatin resistance alters protein expression of Tenascin-C and PAI-1, with little effect on EMT protein expression levels in spheroid structures

**FIGURE 6:**
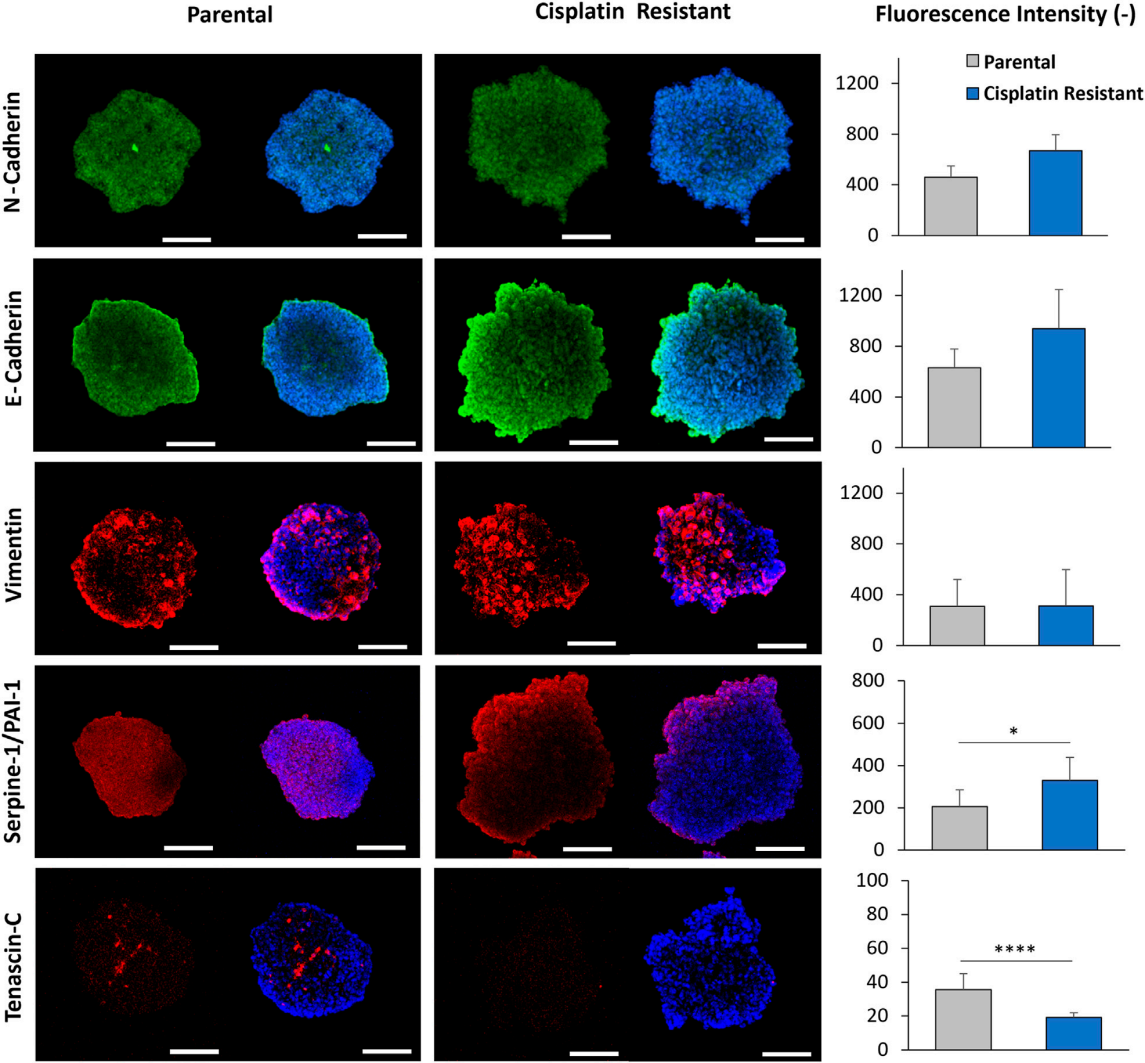
CLSM imaging of 48 h parental and cisplatin-resistant SKOV3 spheroids that were immunofluorescently stained with primary/secondary antibodies to visualize E-cadherin, N-cadherin, vimentin, serpine-1/PA1-1 and tenascin-C. Furthermore, the cells were counterstained with Hoechst to visualize the nucleus (Scale bar 200 µm). Quantification of the fluorescence intensity of E-cadherin, N-cadherin, tenascin-C, vimentin, and serpine-1/PA1-1 markers expressed in the spheroids from the CLSM images is shown. Data is shown as the mean and SD of a minimum of five biological spheroid repeats, statistically analysed as non-parametric data using Mann Whitney test. Significance given as *p < 0.05, ****p < 0.0001.

Due to alterations in gene expression associated with the cytoskeleton and ECM in CR spheroids, the protein expression of EMT markers (E-cadherin, N-cadherin, Vimentin) were examined in P and CR cells and spheroids using immunofluorescence staining (Supplementary Figure S5A and Figure 6). In addition, Tenascin-C was included in the protein expression assays as recent studies have demonstrated that Tenascin-C is not only an important EMT marker in breast cancer but also has been shown to be an important tissue remodelling glycoprotein, promoting proliferation, invasion and angiogenesis, thereby contributing to tumorigenesis and metastasis (Wilson et al., 1996; 1999; Didem et al., 2014; Tucker and Degen, 2022). Counter to the gene expression profiles obtained from P and CR spheroids, quantification of the EMT protein markers in cells alone revealed a significant upregulation of E-cadherin and a significant downregulation of vimentin (p < 0.01), with no significant differences in N-cadherin and Tenascin-C expression between the P and CR cells (p > 0.05; Supplementary Figure S5b). Moreover, quantification of the shape form factor (Azizullah, 2018) used to describe cell morphology did not reveal any significant alterations in cell shape that would be expected during EMT (p > 0.05; Supplementary Figure S5C), where cells would be expected to display a more mesenchymal morphology. The results of the protein assays indicate that EMT transitions are not occurring within CR cells.

The protein expression associated with EMT markers within P and CR spheroid structures (Figure 6), assessed through immunofluorescence staining, did not reveal any significant differences in the fluorescence intensity of E-cadherin, N-cadherin and Vimentin expression between P and CR spheroids (p > 0.05). Western blot analysis confirmed this result as no significant differences in N-cadherin or Vimentin protein expression were determined however, there was a significant decrease in E-cadherin in CR spheroids compared to P spheroids (p < 0.05; Supplementary Figures S6, S7). Interestingly, CLSM imaging revealed a localised increase in E-cadherin expression present on the outermost cellular layers of the proliferate zone in both spheroids, where the intensity of E-cadherin staining appeared to be greater for the more loosely-aggregated CR spheroids. There was also a significant downregulation of Tenascin-C expression observed in CR spheroids through immunofluorescence staining, with expression present only in discrete areas, however, this was not confirmed through Western blot analysis (Supplementary Figure S6). Furthermore, the expression of PAI-1 protein within spheroid structures was assessed (Figure 6; Supplementary Figure S6, S7). The PAI-1 protein, encoded by the gene SERPINE1, has been previously shown to impede cell binding to ECM proteins by blocking the urokinase plasminogen activator receptor (uPAR)/integrin–vitronectin cellular binding mechanism (Hapke et al., 2001; Czekay et al., 2003; Ricciardelli et al., 2016). As the SERPINE-1 gene was the most upregulated gene in CR spheroids from the PCR arrays, PAI-1 protein expression was examined in both spheroids. Quantification of the fluorescence intensity of PAI-1 staining revealed a significant upregulation of the protein (p < 0.05; Figure 6) within the CR spheroids in comparison to P, which was confirmed by Western blot analysis (p < 0.01; Supplementary Figure S6, S7). The altered expression of Tenascin-C and PAI-1 proteins, both involved in cellular and ECM binding mechanisms (Midwood and Orend, 2009; De Laporte et al., 2013; Popova and Jücker, 2022), within CR spheroids highlights their potential role in EOC spheroid architecture.

### Cisplatin resistance influences the adhesion and disaggregation of spheroids, and also cellular invasion, into a basement membrane mimic

**FIGURE 7:**
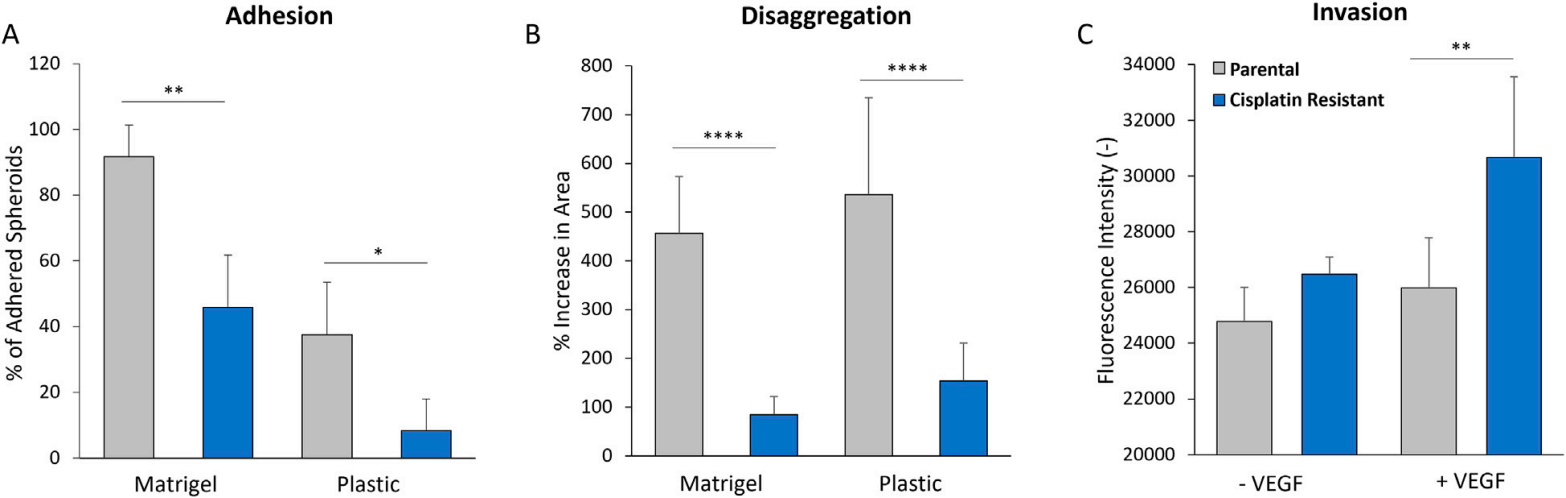
**(A)** Adhesion of 48 h parental and cisplatin-resistant SKOV3 spheroids to uncoated plastic and Matrigel-coated surfaces after 3 h incubation. **(B)** Disaggregation of 48 h parental and cisplatin-resistant SKOV3 spheroids on uncoated plastic and Matrigel-coated surfaces after 24 h incubation, as measured by % increase in spheroid area. **(C)** Invasion of parental and cisplatin-resistant SKOV3 cells through basement membrane-coated transwells in the presence and absence of VEGF, as measured by fluorescence intensity of stained cells in the lower chamber. Data is shown as the mean with a SD of a minimum of six spheroids and a minimum of three biological repeats, statistically analysed as either parametric data using one-way ANOVA test with Tukey’s multiple comparison test or non-parametric data using the Kruskal-Wallis test with Dunn’s multiple comparison test. Significance given as *p < 0.05, **p < 0.01, ***p < 0.001, ****p < 0.0001.

Matrigel, a solubilised basement membrane hydrogel preparation rich in ECM proteins, such as laminin and collagen IV, was used to model the ECM layer exposed by mesothelium clearance in the peritoneal lining (Kenny et al., 2007; Lengyel, 2010; Liao et al., 2014; Al Habyan et al., 2018). The attachment and subsequent disaggregation of the CR and P spheroids on the Matrigel surfaces was examined, with a plastic surface control to determine the intrinsic adherence properties of the spheroids themselves. The CR spheroids were significantly less adherent after 3 h incubation on both Matrigel (mean values: 91.7% vs. 45.9%; p < 0.01) and plastic surfaces (mean values: 37.5% vs. 8.33%; p < 0.05) compared to P spheroids (Figures 7A, B). The CR spheroids also showed significantly less disaggregation after 24 h incubation on both the Matrigel (mean values: 456% vs. 84%; p < 0.001) and the plastic surface (mean values: 536% vs. 153%; p < 0.001) when compared to P spheroids. The contrasting morphology and mechanical properties of the CR spheroids seem to favour altered surface interactions, which may be linked to differential invasive potential acquired with cisplatin resistance.

The 2D cell invasion assay, with inclusion of ECM basement membrane (Figure 7C), was used to model single cell invasion through the ECM layer in the peritoneal lining. High levels of vascular endothelial growth factor (VEGF) expression have been measured in the serum, ascites and tumors of ovarian cancer patients (Zhang et al., 2006; Lengyel, 2010), where it is thought to enhance ovarian cancer cellular invasion through the basement membranes (Wang et al., 2008). As such, VEGF was added into the cell invasion assay as a positive control. Whilst there was no significant difference in the invasion of P and CR cells through the ECM layer in the absence of VEGF (p > 0.05), the CR cells in the presence of VEGF demonstrated a greater capacity to invade through the ECM layer in comparison to the P cells (mean values of fluorescence intensity: 25,985 vs. 30,660; p < 0.01).

## Discussion

Unravelling the role of ovarian cancer multicellular aggregates in drug resistance and routes to metastasis, requires multi-resolution analysis of their molecular, cellular and tissue-like properties (Lengyel, 2010; Chowanadisai et al., 2016; Vyas et al., 2019; Pisano et al., 2021). Indeed, the molecular and biophysical properties of such metastatic units are becoming more widely established, due to the role of biophysical interactions in driving aggregate formation, interaction with other cell types and subsequent metastasis (Blumlein et al., 2017; Andolfi et al., 2019; Krieg et al., 2019; Vyas et al., 2019; Abidine et al., 2021; Boot et al., 2021). To decipher these biophysical mechanisms, we have developed a comparative *in vitro* 3D spheroid model based on P and CR SKOV3 cell lines.

*In vivo*, ovarian cancer cells exfoliate from the primary tumour as either single cells or metastatic units and circulate the peritoneal space via ascites fluid diffusion (Kenny et al., 2007; Liao et al., 2014; Chowanadisai et al., 2016; van Baal et al., 2017; Hedemann et al., 2018). The ability of multicellular aggregates to form and withstand the shear forces acting in the ascites fluid within the peritoneal cavity is postulated to be linked to their biophysical properties (Lengyel, 2010; Chowanadisai et al., 2016; Novak et al., 2018; Vyas et al., 2019). A study by Ip et al. (2016) found that SKOV3 spheroids exposed to clinically-relevant low shear forces (which act in the malignant ascites) resulted in the expression of EMT and cancer stem cells (CSC) markers, whilst also demonstrating chemoresistance to cisplatin and paclitaxel. Importantly, additional mechanisms of resistance can occur within the multicellular aggregate structure which contribute to enhanced drug tolerance (Han et al., 2021), with these functions related to their biophysical and mechanical properties (Krieg et al., 2019). The tightly packed cellular spheroid structure with increased expression of ECM proteins may impose diffusional limits to the mass transport of therapeutic agents into the structure (Lengyel, 2010; Mehta et al., 2012; Jaiswal et al., 2017; Vyas et al., 2019), while the presence of hypoxic cells in large spheroids may also increase resistance to therapy due to altered oxygen and nutrient diffusion gradients (Vyas et al., 2019; Han et al., 2021; Refet-Mollof et al., 2021). For example, the chemoresistance to doxorubicin (DOX) was 50 times higher in MCF-7 cellular spheroids compared to 2D culture (Chowanadisai et al., 2016), while spheroids formed from human lung carcinoma (A549) cells were 6,600 times more resistance to vinblastine compared to monolayer cells, as measured by a IC50 assay (Desoize and Jardillier, 2000). Such studies highlight the importance of the biophysical structural arrangement of multicellular aggregates in relation to drug resistance and cancer treatment (Vyas et al., 2019).

Through the development of specific AFM protocols, our study has demonstrated, for the first time, that spheroids formed from CR cells possess altered morphologies and elastic properties when compared to P spheroids. Optimisation of the AFM protocol also showed that the measurements of elasticity, indentation and probe adhesion were dependent on the indentation force applied to the complex, rough morphological surface of the spheroid model however, there was limited dependence of tip frequency on the biophysical parameters determined by AFM. The Young’s modulus of both the P and CR SKOV3 spheroids examined in this study ranged from 0.27–1.65 kPa (depending on experimental conditions) which is in line with other spheroid mechanical studies. Guillaume et al. (2019) used AFM with sharp-tip probes to reveal variations in surface topography and elasticity (2–10 kPa) of colorectal carcinoma spheroids while other studies using complementary techniques demonstrated elastic moduli values of 13–500 Pa for HEK cell spheroids (Blumlein et al., 2017) and revealed that non-malignant epithelial breast cell spheroids (MCF 10A) were significantly stiffer than spheroids formed from two cancerous (T47D and BT474) breast cell lines (230 vs. 1250 Pa; Jasiwal et al., 2017). While the cellular cytoskeleton has been commonly identified as the major mechanical structure of cells (Pegoraro et al., 2017), it is the ECM, a highly complex fibrous construct of proteins (collagen, fibronectin) and polysaccharides (hyaluronan and glycosaminoglycan), which provides the structural and mechanical support required for spheroid and tissue integrity (Jaiswal et al., 2017; Han et al., 2021; Tucker and Degen, 2022). Indeed, Vyas et al. (2019) performed an AFM depth-dependent indentation profiling study, revealing nanomechanical heterogeneity in the proliferation zone of lung carcinoma spheroids, due to the complex agglomerate of cells and collagen-based structures within the ECM. This is akin to what is found in ovarian cancer spheroids in this study, with altered elasticity observed in CR SKOV3 structures.

To understand the impact of cisplatin resistance on multicellular aggregate architecture, genes involved in the ECM and cytoskeleton were profiled. There was altered expression of collagen, fibronectin and matrix metalloproteinases (MMP’s), with an upregulation in vimentin and a downregulation in E-cadherin in CR spheroids compared to P spheroids, which is indicative of at least a partial EMT process. Interestingly, EMT in ovarian cancer cell lines has been implicated in promoting resistance to chemotherapeutic agents, through mechanisms such as higher efflux of the drug, the presence of β-Tubulin variants and changes in the MAPK/ERK pathway (Loret et al., 2019). In EOC, cells initially deattach from the primary ovarian tumour through cellular EMT which loosens intercellular adhesions by downregulating the membrane glycoprotein E-cadherin (located at the cell adherent junctions), upregulating other cadherins (N-cadherin, P-cadherin), changing integrin expression and upregulation of proteolytic pathways (Lengyel, 2010). The initial formation of multicellular aggregates within the peritoneal cavity then occurs by integrin-mediated attachment to ECM molecules, followed by increased E-cadherin interactions which results in compact multicellular aggregate structures (Han et al., 2021). Studies have shown that cells expressing high E-cadherin form compact spheroids (Han et al., 2021). Even though the results achieved from the protein expression studies in both cells and spheroids did not demonstrate any EMT transitions in CR compared to P cell lines, it was observed that E-cadherin staining present on the outermost cellular layers of the proliferate zone appeared to be greater for the more loosely-aggregated CR spheroids. Interestingly, a study by Matte et al. (2016) also found that there was an unexpected correlation between high expression of E-cadherin and less compact *in vitro* ovarian cancer spheroids. Therefore, it is unlikely that EMT is responsible for the more loosely-aggregated, softer CR EOC spheroid structure.

Tenascin-C is a large extracellular glycoprotein which is an important EMT marker in breast cancer and has been implicated in the mechanical properties of both heart and cartilage tissue (Midwood and Orend, 2009; Cho et al., 2015). In this study, CR SKOV3 spheroids showed significantly reduced expression of tenascin-C compared to P spheroids in immunofluorescence assays. In EOC, the levels of tenascin-C are significantly higher than in non-cancer controls (Didem et al., 2014), where a study by Wilson et al. (1996) identified that tenascin-C was significantly overexpressed in the stroma of malignant ovarian tumours when compared to benign ovarian tumours. Interestingly, tenascin-C also possesses the ability to interact directly with a number of cell types through binding to cellular receptors (integrins, heparan sulfate proteoglycan) and ECM ligands (fibronectin, perlecan, versican; De Laporte et al., 2013). This multi-binding capacity of tenascin-C may provide this glycoprotein with crosslinking functions which may modulate spheroid architecture (Midwood and Orend, 2009; Popova and Jücker, 2022). Even more so, AFM force measurements have previously demonstrated that tenascin-C is an elastic ECM protein, where a single molecule of tenascin-C could be stretched to several times its resting length (Oberhauser et al., 1998). The reduced expression of tenascin-C in the CR SKOV3 spheroids, determined through immunofluorescence, may have contributed to alterations in elasticity and the more loosely aggregated spheroid structure. However, the quantity of tenascin-C protein expression determined through both immunofluorescence and Western blot analysis was small in comparison to the other markers.

In further investigations, PAI-1 protein expression was also examined in both CR and P spheroids to begin to understand the mechanisms which result in the altered biophysical properties of CR spheroids. In the gene arrays, SERPINE1 was the most upregulated gene in CR spheroids (PAI-1 protein is encoded by the gene SERPINE1), where both immunofluorescence and western-bolt assays confirmed significant upregulation of PAI-1 protein. Increased levels of PAI-1 have been shown to be associated with reduced ovarian cancer survival (Nakatsuka et al., 2017). Also, studies have shown when PAI-1 protein levels are elevated, PAI-1 can impede cellular binding to the ECM protein vitronectin by disrupting the interaction between the cell membrane receptor uPAR (together with integrins) and vitronectin (Hapke et al., 2001; Czekay et al., 2003; Ricciardelli et al., 2016). The affinity of PAI-1 to the NH_2_-terminal somatomedin B domain of vitronectin is much higher than the affinity of uPAR to vitronectin, so the PAI-1 protein can competitively inhibit the uPAR-dependent attachment of the cell to vitronectin. This “deadhesive” ability of PAI-1 may explain why high PAI-1 levels are associated with poor prognosis in human metastasis disease (Hapke et al., 2001; Czekay et al., 2003; Ricciardelli et al., 2016). Studies have confirmed the presence of vitronectin, together with α_v_ and β_1_ integrins, on the SKOV3 cell surface (Cruet-Hennequart et al., 2003; Carduner et al., 2013) and SKOV3 cells have been shown to be significantly less adhesive to vitronectin-coated surfaces in the presence of anti-uPAR antibodies (Minopoli et al., 2019). These studies highlight that increased PAI-1 protein in the CR SKOV3 spheroids could be disrupting the uPAR/integrin–vitronectin cellular binding mechanism, resulting in reduced intracellular adhesion within the spheroid. This reduction in intracellular adhesion within the CR spheroid could therefore, result in a significant alteration in spheroid elasticity, with a more loosely aggregated spheroid structure. Further research utilising gene knockouts targeting SERPINE 1 (PAI-1), together with uPAR antibodies and vitronectin cellular staining, would confirm that increased PAI-1 protein expression in CR spheroids results in reduced intracellular adhesion.

In metastasis, there is the dissemination of the cancerous multicellular aggregates within the peritoneal cavity, by the ascites, to the secondary site of the peritoneum (Novak et al., 2018), where the multicellular aggregates encounter the mesothelium. Kenny et al. (2007) demonstrated *in vitro* that an intact mesothelial cell layer will efficiently delay ovarian carcinoma cell adhesion, suggesting delayed cell attachment and invasion. Interestingly, Heyman et al. (2008) revealed that the adhesion of ovarian cancer cells to mesothelial cells was significantly inhibited through using anti-vitronectin, -αv-integrin and uPAR-blocking antibodies, suggesting that elevated levels of the PAI-1 protein could impede ovarian cancer cell binding to the peritoneal mesothelium through the protein’s interaction with vitronectin and its receptors. There is evidence to demonstrate that ovarian multicellular aggregates use myosin-generated force to clear the mesothelium, allowing the aggregates to adhere and invade through the underlaying ECM layer (basement membrane composed of collagen I and IV, fibronectin and laminin; Al Habyan et al., 2018; Iwanicki et al., 2011; Lengyel, 2010; Liao et al., 2014). Once the tumour cells have metastasized into the peritoneum, the cells experience mesenchymal-epithelial transition (MET) which allows them to sustain fast growth and respond to paracrine growth factors (Lengyel, 2010; Boot et al., 2021). To understand if the altered biophysical properties of multicellular aggregates through the acquisition of cisplatin resistance influence the metastasis process, we examined how the CR and P spheroids adhered, disaggregated and invaded through an ECM mimic. The more loosely-associated, less compact CR spheroids, with a greater intensity of E-cadherin present in the spheroid outer cell layers, were slower to adhere and disaggregate across Matrigel and plastic surfaces in comparison to P spheroids. However, the presence of VEGF enhanced the ability of CR cells to invade through an ECM mimic compared to the P cells, suggesting that once the CR cells have migrated from the spheroid, they possess an enhanced ability to invade through the ECM lining. To fully understand the cell clearance and dissemination mechanisms of CR multicellular aggregates across the mesothelium *in vivo*, a more complex *in vitro* co-culture model of a mesothelium cell layer grown on top of an extracellular mimic would have to be used (Kenny et al., 2007).

This study highlights that CR spheroids, with a less compact and mechanically weaker architecture, may be slower in the initial attachment and subsequent disaggregation across the peritoneal ECM lining in comparison to P spheroids. Altered protein expression of E-cadherin, Tenascin-C and PAI-1, alongside altered collagen and MMP gene expression profiles, signified a more loosely packed outer cellular layer in the CR spheroids and a more aggressive invasive phenotype compared to the P cells. This interdisciplinary investigation highlights new multi-parameter insights to ovarian cancer derived multicellular aggregate formation, adhesion and invasion, in the context of chemoresistance and metastasis, which may be beneficial in the development of new targeted treatment strategies to combat high grade serous ovarian cancers (Lengyel, 2010; Chowanadisai et al., 2016; Vyas et al., 2019; Pisano et al., 2021).

## Conclusion

This study has revealed distinct differences in the biophysical properties and gene/protein expression of CR spheroids in comparison to P spheroids, which may influence the metastatic potential of the ovarian cancer spheroids when encountering the peritoneal lining. This greater mechanistic understanding of the relationship between drug resistance and spheroid architecture highlights key proteins which could be responsible for altered metastatic progression that may occur in ovarian cancer patients that present with cisplatin resistance.

## Data Availability

The original contributions presented in the study are included in the article/Supplementary Material, further inquiries can be directed to the corresponding authors.
