# Supplementary figures and images for: Cisplatin resistance alters ovarian cancer spheroid formation and impacts peritoneal invasion
Source: Front Cell Dev Biol. 2025 Feb 5;13:1450407. doi: 10.3389/fcell.2025.1450407 (PMC11836028; doi:10.3389/fcell.2025.1450407)

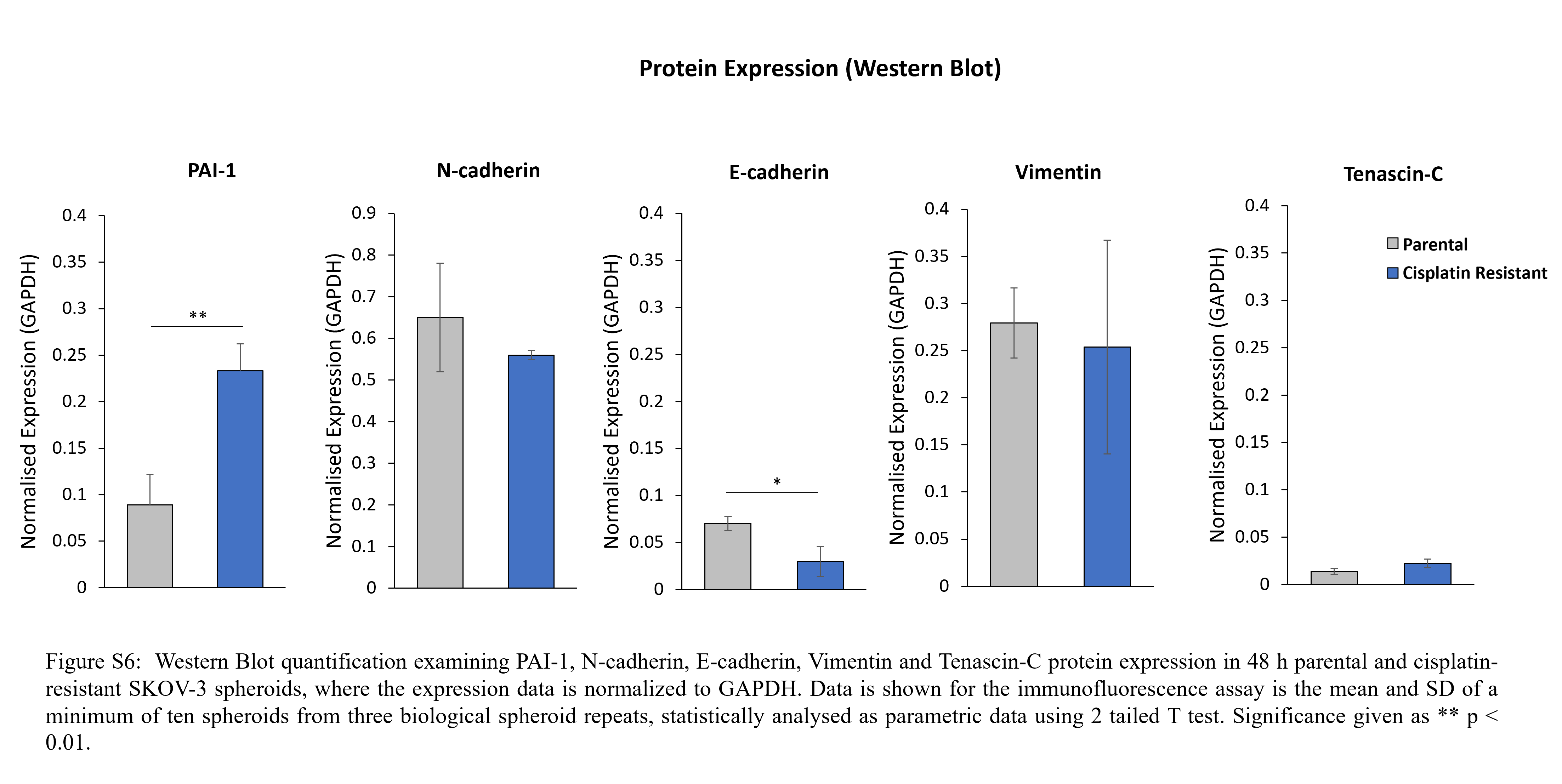

Supplement: Supplementary file 1 [file Image6.tif]

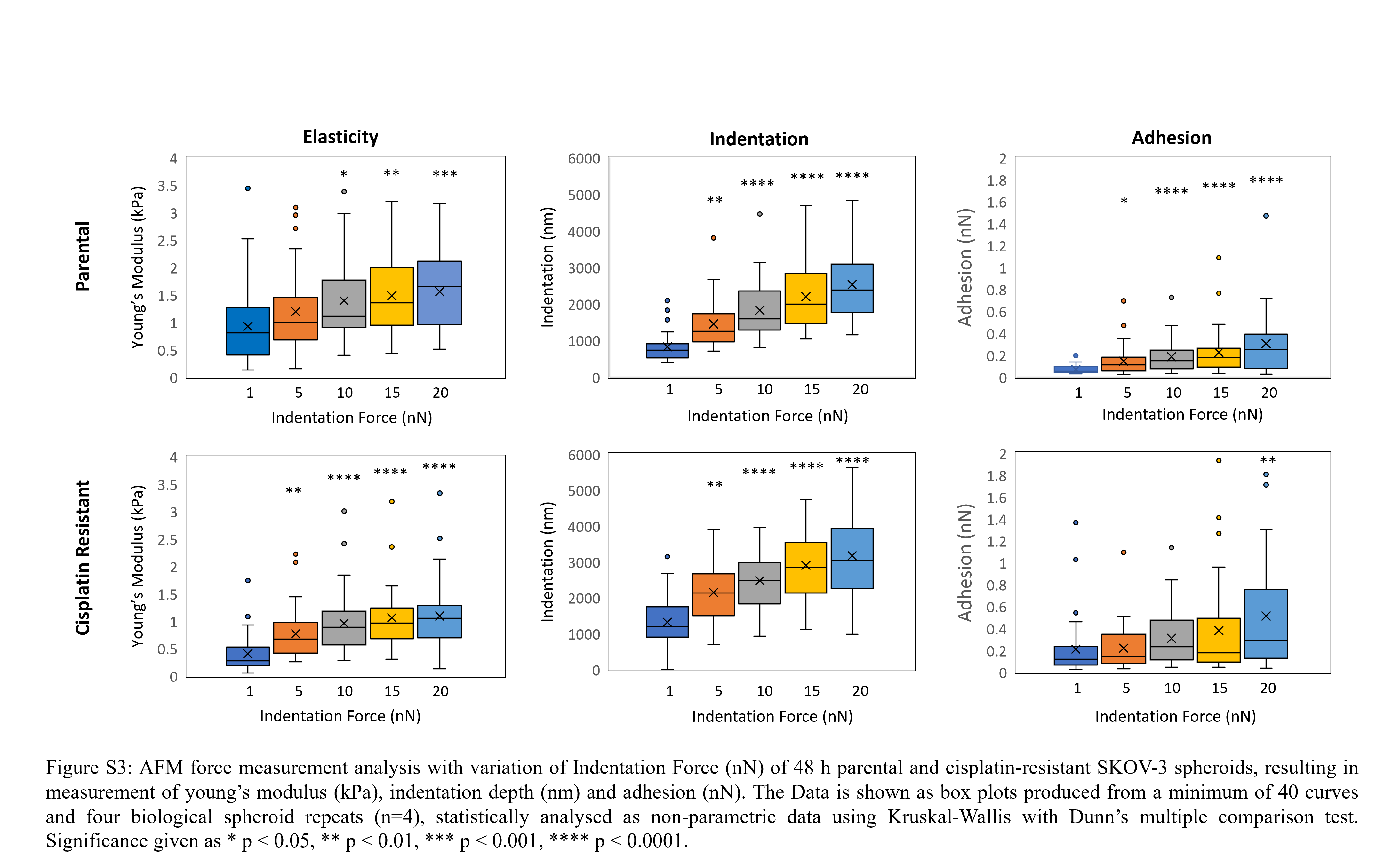

Supplement: Supplementary file 2 [file Image3.tif]

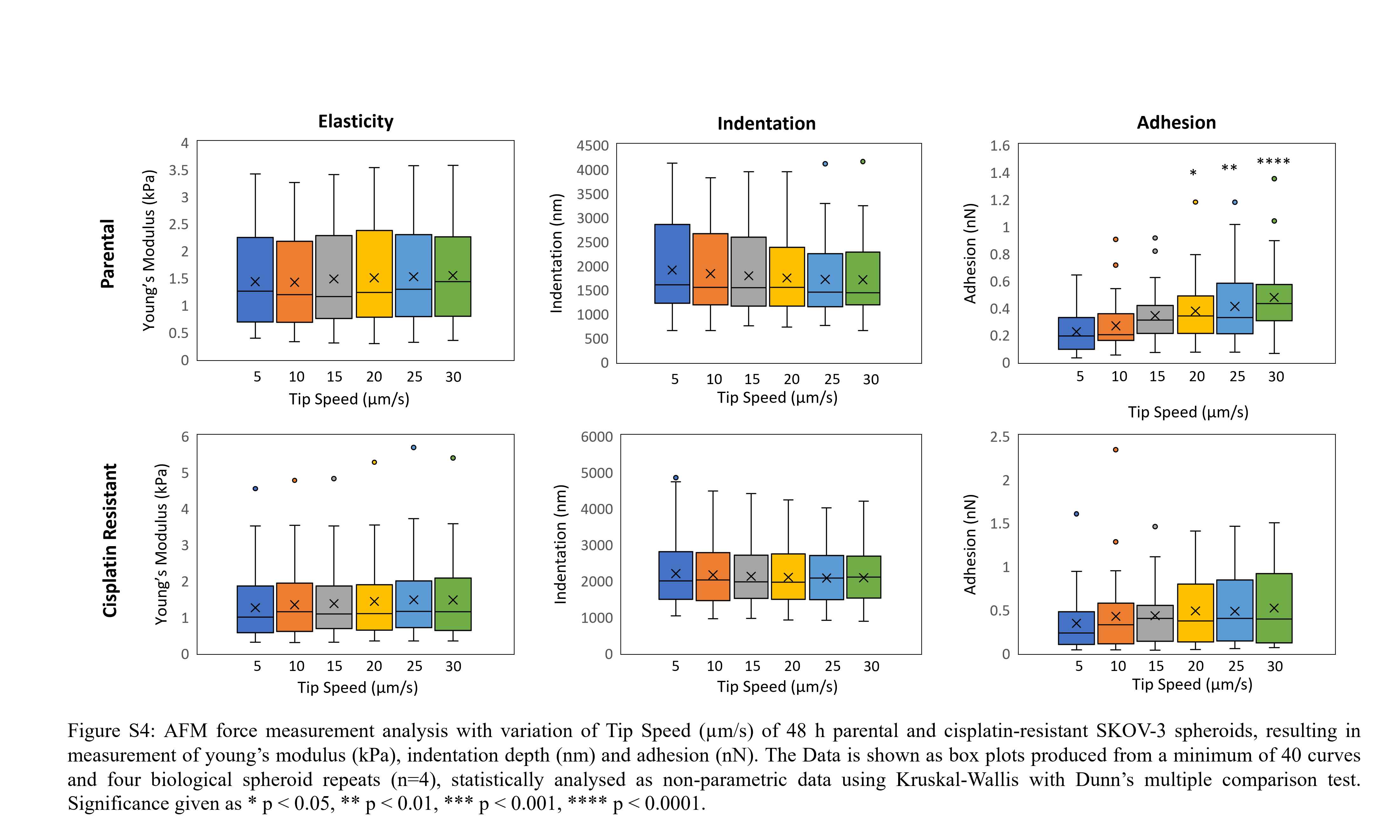

Supplement: Supplementary file 3 [file Image4.tif]

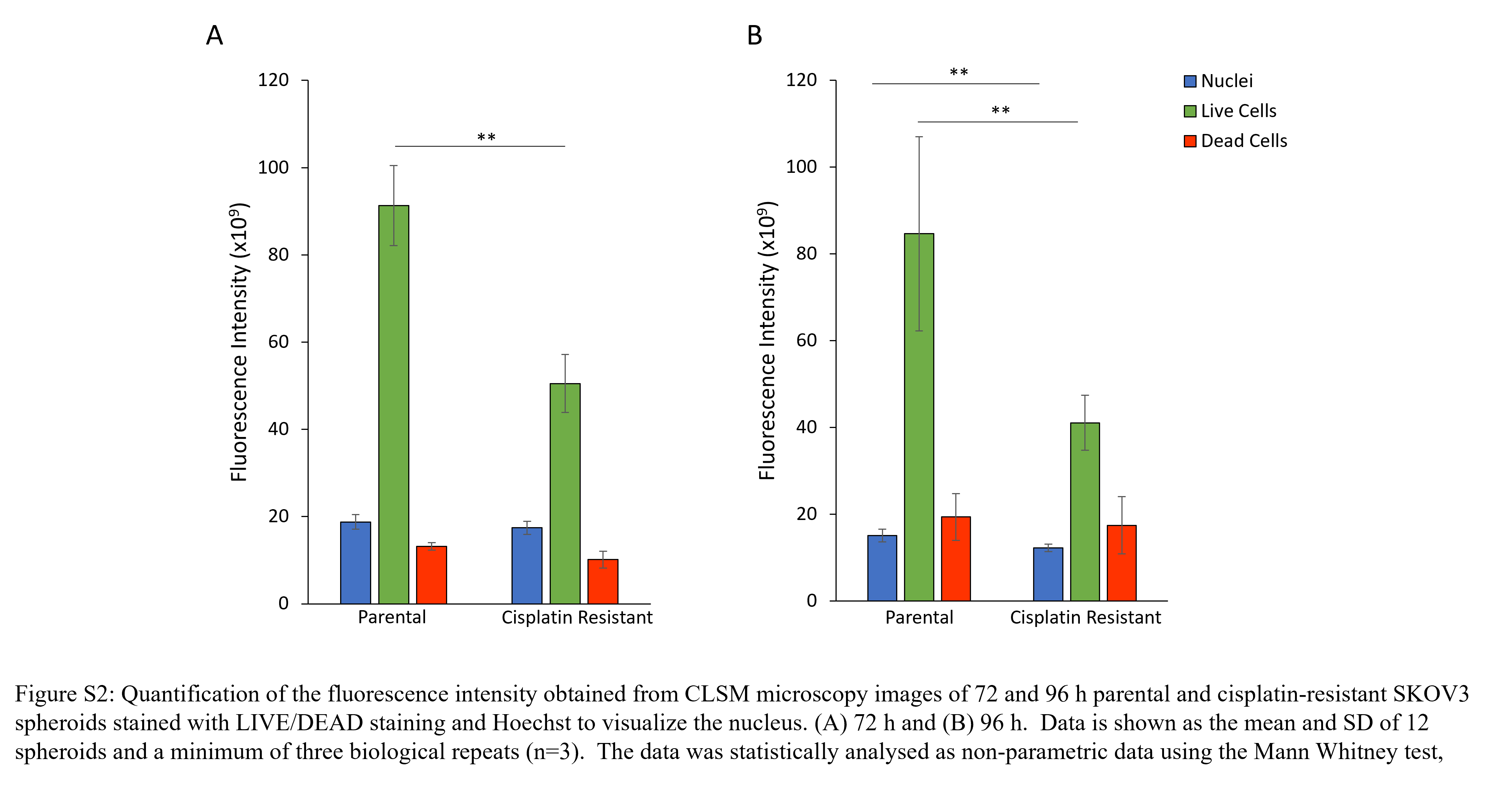

Supplement: Supplementary file 4 [file Image2.tif]

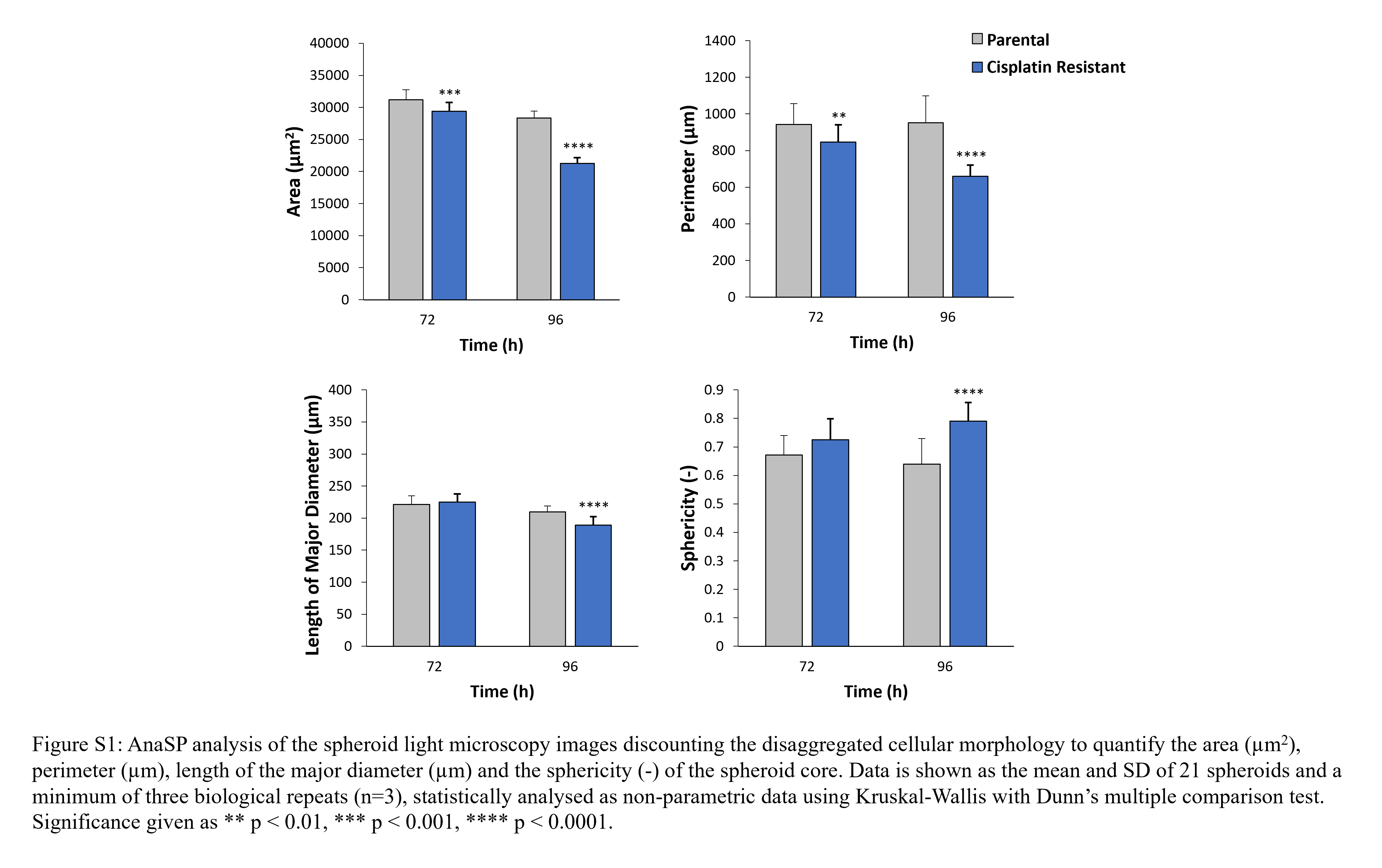

Supplement: Supplementary file 5 [file Image1.tif]

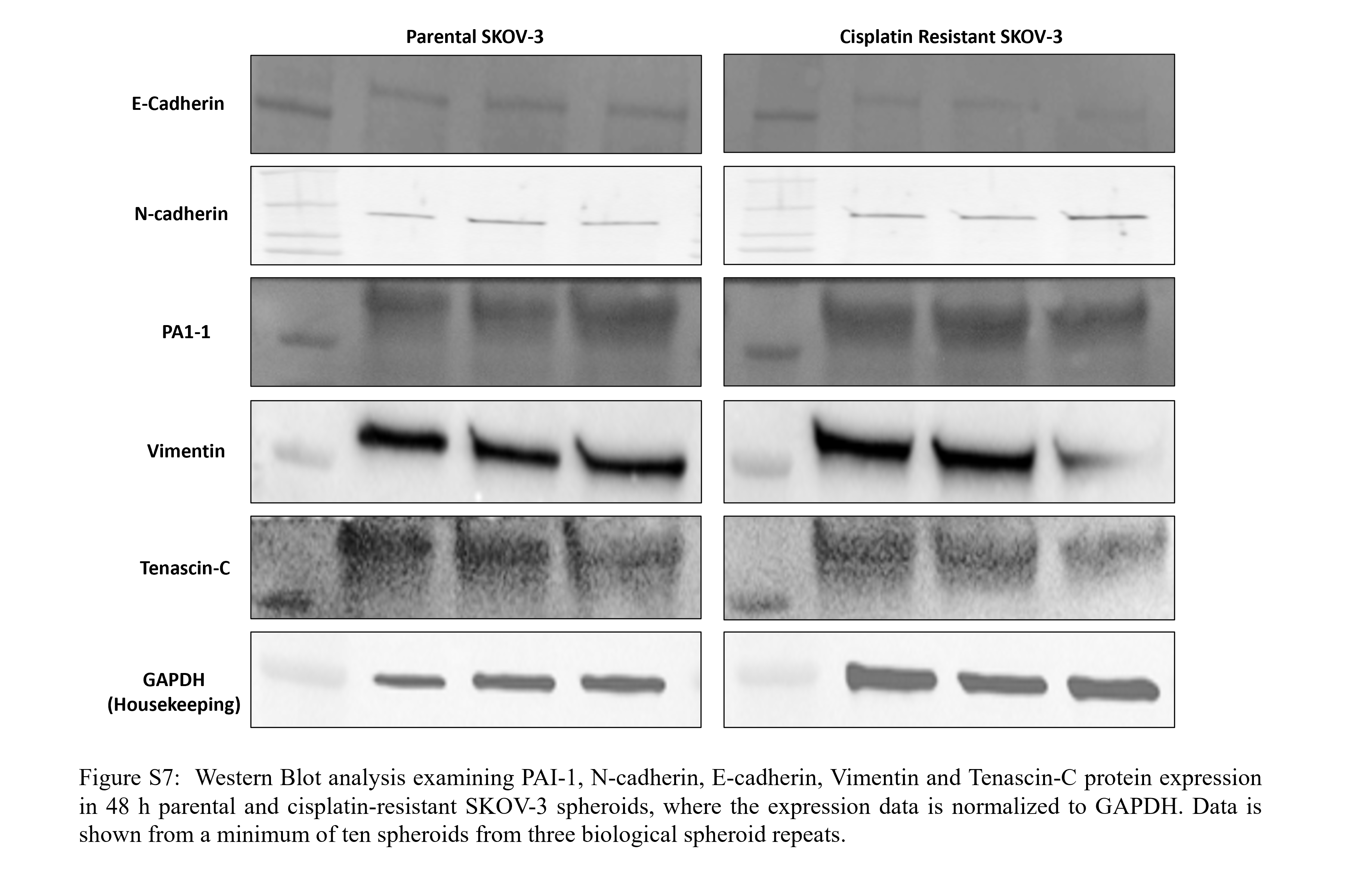

Supplement: Supplementary file 6 [file Image7.tif]

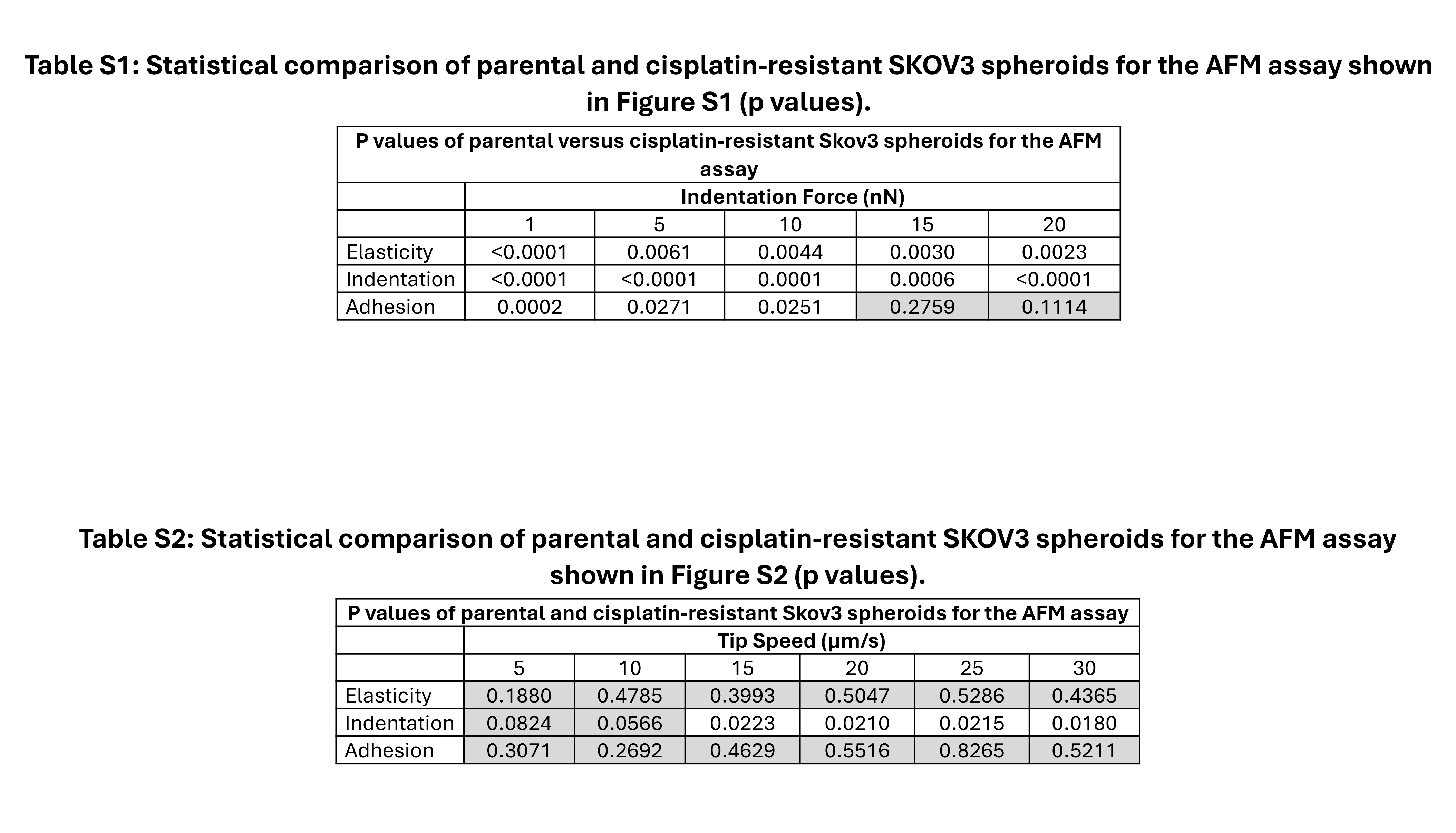

Supplement: Supplementary file 7 [file Image8.tif]

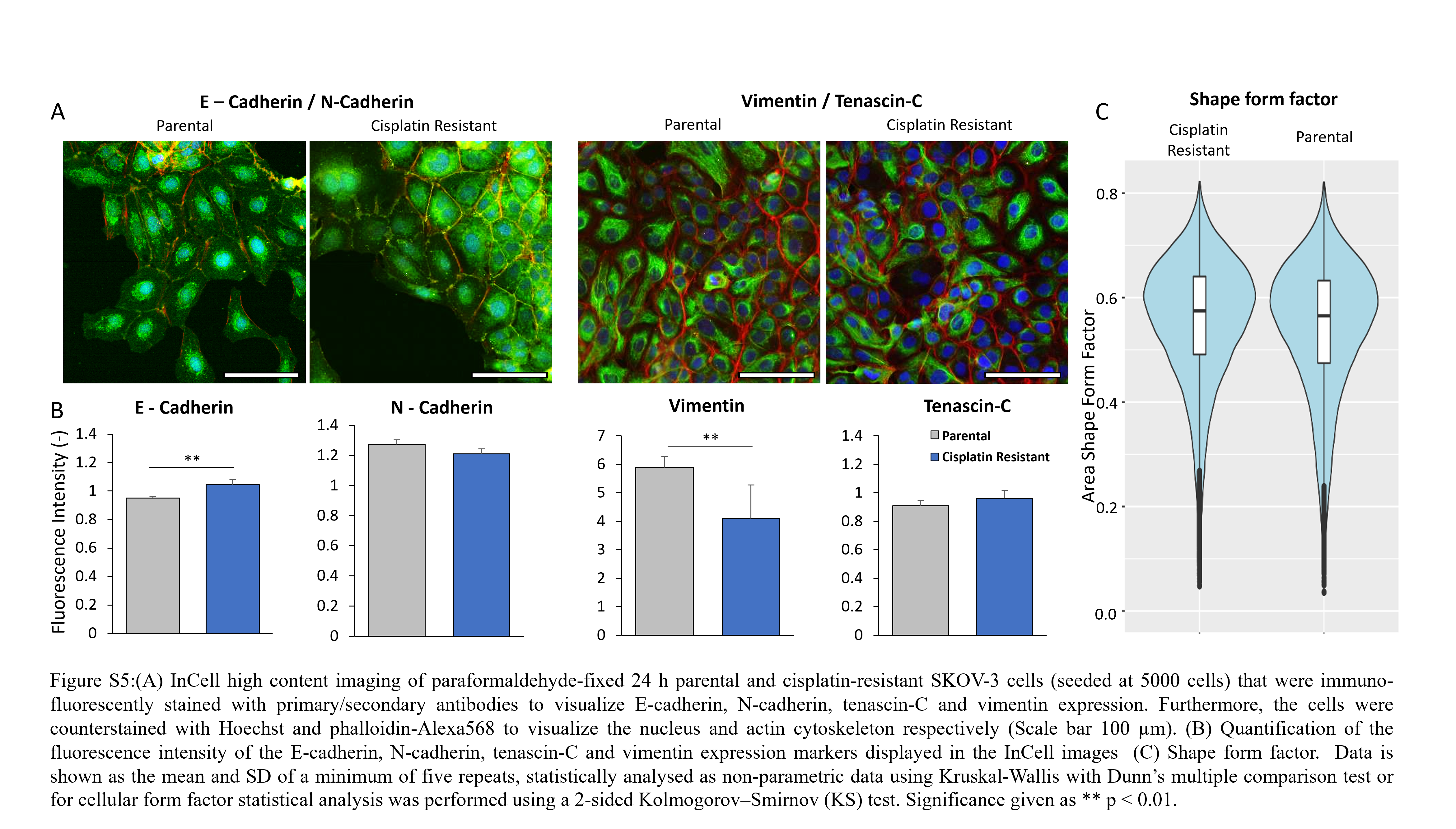

Supplement: Supplementary file 8 [file Image5.tif]
